# International Humanitarian Law and the Immunity of Hospitals in Gaza

**DOI:** 10.1111/bioe.13433

**Published:** 2025-06-15

**Authors:** Zohar Lederman

**Affiliations:** ^1^ Department of Emergency Medicine University of Hong Kong Hong kong Hong kong; ^2^ Centre for Medical Ethics and Law, University of Hong Kong Hong kong Hong kong

**Keywords:** combatants, Gaza, Geneva Convention, hospitals, immunity, Israel

## Abstract

International Humanitarian Law (IHL), specifically Article 18 of the IV Geneva Convention, affords special protection to civilian hospitals. This special protection is waived, however, under certain circumstances specified in Article 19. Such conditions to waive the special protection of hospitals are now being used by Israel to justify the attack on civilian hospitals and healthcare institutions in Gaza. This paper critically evaluates Article 19 and the conditions for the removal of the immunity of hospitals in general and in the specific case of Gaza. The substance and language of Article 19 are found to be flawed in this case. The paper thus argues that Article 19 should be revised to better reflect the special protection hospitals generally and in Gaza specifically should have. This paper is primarily geared at fellow bioethicists who wish to contribute to and lament the injustices occurring in Gaza and elsewhere but are unsure as to how ethical arguments may do so. This paper also addresses international law scholars, inviting further commentary on a novel and ambitious ethical argument to revise long‐standing international law. Additionally, the paper is a call to the wider, global public and healthcare providers to actively condemn unjust attacks on healthcare in Gaza and elsewhere in the world. Lastly, the paper is written in a meager attempt at standing in solidarity with the People in Gaza and elsewhere whose healthcare systems are being targeted by unjust governments.

## 1 Introduction

Healthcare institutions, namely clinics, ambulances, and hospitals, are commonly targeted during armed conflicts. Only since the early 1990s, healthcare institutions have been targeted in Bosnia, Croatia, Burundi, Rwanda, Somalia, Yemen, Syria, Chechnya, Sri Lanka, Afghanistan, the Central African Republic, Chechnya, Turkey, Liberia, Myanmar, and the Occupied Palestinian Territory [1, 2, 3]. Yet, apart from a few exceptions, bioethicists have been mostly silent about the ethics of war in general and specifically about the targeting of healthcare institutions. Some have even condoned such attacks [4]. While focusing on the ongoing hostilities in Gaza, this paper poses an urgent call on fellow bioethicists to engage these issues to a larger extent and defend the right of persons worldwide to access healthcare, which is and should be one of our main goals.

The atrocious attack by Hamas on Israel on October 7th has resulted in the death of 1161 Israelis and people of other nationalities [4, 5]; 251 Israelis and non‐Israelis were kidnaped [5]. It will be remembered as one of the darkest days in Jewish history. Israel has retaliated in kind, killing 48,388 people—mostly children and women—and injuring 111,803 people in the Gaza Strip (hereon Gaza) as of February 28, 2025 [6]. Thousands are reported missing or under the rubble [7]. More than 8000 cases of chickenpox were documented, as well as more than 760,000 cases of acute respiratory infections, more than 415,000 cases of diarrhea, and more than 90,900 cases of scabies and lice.

Israel has bombarded the Indonesian hospital, reportedly without warning, killing at least 12 people [8], and has twice raided the Al‐Shifa hospital, the largest hospital in the Gaza Strip [9]. Israel has declared its intent to bombard Al Shifa hospital several times in the past, but may have been dissuaded by increasing international pressure. Alternatively, Al Shifa may have been spared because of the Israeli intelligence that hostages were held there [10]. In February 2024, Israel bombarded Nasser Hospital in Rafah, the second largest hospital in Gaza, taking it out of service and killing patients who were inside [11].

The ostensible reason for the bombing of healthcare facilities in Gaza is strategic. Israel knows about the existence of tunnels underneath at least some of the hospitals, simply because it built them [12]. The Israeli Defense Forces (IDF) and Israeli politicians have long alleged that these facilities and the civilians in them were being used as human shields by Hamas combatants who expect immunity while devising strategic plans, firing at Israeli soldiers, or using these facilities to hide hostages. The IDF has specifically alleged that Hamas headquarters are located underneath the Al Shifa hospital, and in its raid has indeed uncovered a complex network of fortified tunnels, numerous weapons, and combat equipment allegedly used by Hamas, as well as evidence of hostages being held there [13, 14]. The director of Al Shifa, under duress, also admitted that Hamas has used the hospital for strategic purposes and that he himself was recruited by Hamas [15]. At the same time, these hospitals have been treating thousands of patients, including newborns in incubators (some were transferred to Egypt), dialysis patients, and patients too severely injured by Israeli bombings to mobilize. The targeting of medical units and infrastructure by Israel during its five major military campaigns in Gaza has indeed been defined as medical lawfare—the cynical use of international law to justify the targeting of civilian medical targets because Hamas itself is breaking international law by using these facilities for military purposes and using civilians as human shields [16].

This article aims to ethically evaluate existing IHL that arguably allows the targeting of civilian medical facilities under specific circumstances. It finds such circumstances not nuanced enough and hence IHL to be ethically questionable in this case. The article also argues that even in its current version, IHL does not allow the targeting of hospitals in Gaza.

Several strategies may be employed to explain, solidify, and/or increase the moral and legal immunity of hospitals in Gaza and elsewhere. A recent publication, for instance, introduces bioethical principles to supplement IHL in evaluating the long‐term effects of war on public health [17]. At least two of these principles may apply here.

*Health Justice* recognizes a deontological duty to distribute health‐related benefits and duties fairly, especially among vulnerable populations, for example, women. Under this principle, hospitals should be protected as they provide care for these vulnerable populations. This principle, however, may be under‐theorized and overly simplistic. First, assuming by default that women are a vulnerable population during war does not withstand the test of reality [18]. Second, this principle does not specify who should ensure the fair distribution of health benefits and burdens. Third, the principle seems to be blind to the actual needs and wishes of these so‐called vulnerable populations. A woman in a place X may wish to have access to healthcare in place X, which she considers to be her home, rather than place Y, into which she was forcefully deported. There is nothing in the principle as it is currently stated that can accommodate that wish and oblige other parties to respect that wish.

*Dignified Lives* may also be used to protect people's right to access healthcare. This principle aims to ensure a minimal threshold of capabilities, including a capability to be healthy, which in turn requires access to social determinants of health, for example, healthcare. But again, this principle is under‐theorized and lacks nuance. The principle does not hold any party accountable and is thus scarcely operationalized. An attacking party may reasonably claim that hospitals have been used as a military outpost, and as such, it should have no obligation to ensure access to healthcare in the long run to civilians of the attacked party. There is nothing in the principle as it is currently stated to hold the attacking party responsible and/or accountable.

Other accounts have relied on IHL to more rigorously defend healthcare institutions [1], and the analysis here follows their footsteps. It adds novelty first by focusing on the current situation in Gaza, and second by actually challenging existing IHL. At the same time, however, the argument here may readily apply to other contexts as well.

Importantly, the argument articulated here is an ethical argument made by an ethicist, not a legal argument made by legal experts. Relying on IHL to make an ethical argument—that hospitals in Gaza should not be targeted—requires an implicit assumption to avoid an is‐ought logical fallacy. Paradoxically, the ethical argument articulated here also makes a case for the revision of IHL. What is the implicit assumption, then, and how can reliance on IHL in one case align with a call to revise it in another? Recapitulating and elaborating a discussion made elsewhere [19], the implicit assumption here is that IHL mirrors, or is grounded in ethical truths. These ethical truths are that humans have an inherent and equal moral worth, elsewhere defined as “a right to equal concern and respect” [20], and that this entails certain fundamental entitlements from other individuals or a community. In other words, humans have moral rights that are independent of any positivist law [20].

Mirroring these ethical truths means that IHL, specifically the Geneva Conventions and their accompanying commentaries, may be considered as authoritative ethical references. IHL is authoritative because it has been drafted and revised over a long period of time, by many different authors, and tested against many wars. It is also authoritative because its explicit underlying values correspond to what I define as moral truths [19]. “Authoritative” does not mean IHL is always right, but it may mean that it is more likely to be right than not, or at least it could be used as a stepping stone to make an initial argument to invite further deliberation and commentary. At the same time, based on a procedure of a wide reflective equilibrium (WRE) [21],^1^ IHL may and should be rigorously and continuously scrutinized and challenged whenever novel, unforeseen challenges arise or whenever its real‐life application seems counterintuitive.

RWE assumes the existence of three layers of beliefs or tentative commitments: considered moral judgments, moral principles, and relevant background theories. These three layers constantly communicate with one another, such that one dictates and is at the same time scrutinized by the other. Relevant background theories, such as a theory about human psychology or about justice between humans across generations and geographical areas, dictate and are evaluated against moral principles, such as John Rawls' Difference Principle or Mill's Harm Principle. These principles then guide moral judgments in specific cases. At the same time, however, moral judgments in specific cases may challenge moral principles and, directly or indirectly (meaning, by first challenging moral principles), relevant background theories.

The ethical analysis here similarly proceeds in two directions: up‐bottom, in relying on background theories and/or moral truths to produce moral principles and then specific moral judgments; and bottom‐up, in allowing moral judgments to challenge and revise moral principles and background theories (the exact content of moral truths can also be questioned epistemologically). Applying then the idea of pre‐legal human rights to IHL and following other philosophical accounts of the moral underpinnings of IHL and human rights law [22, 23], I perceive IHL as a set of moral principles that is grounded in a background moral theory of humanism and the equal moral value and hence respect toward all human beings. In the words of Michael Walzer, IHL corresponds and must correspond to our idea of what is right:

It is probably true that any limits will be useful here, so long as they are in fact commonly accepted. But no limit is accepted simply because it is thought that it will be useful. The war convention must first be morally plausible to large numbers of men and women; it must correspond to our sense of what is right. Only then will we recognize it as a serious obstacle to this or that military decision, and only then can we debate its utility in this or that particular case [24].

Below, I explain why these principles, when applied to the current context of Gaza, should prevent Israel from targeting hospitals. Concomitantly, I rely on reasonable ethical considerations to challenge these principles. Even though I do not explicitly argue for it here, I would also suggest that these moral considerations better align with the background moral theory, thus strengthening the challenge.

## 2 Israel and International Humanitarian Law

Protected by the veto power of the United States in United Nations' Security Councils' meetings, and benefiting from the unwavering support of several governments, for example, Germany [25], Israeli governments have been accused of violating IHL many times in the past [26]. Israeli governments have specifically been accused of targeting healthcare institutions and personnel [27].

During the current war, the United Nations (UN) Secretary‐General invoked the rarely used Article 99 to urgently convene the UN Security Council in the face of a catastrophic situation in Gaza. The Council met on December 8, 2023 and voted on a resolution calling for an immediate humanitarian ceasefire, only to be vetoed by the United States. The US abstained on another resolution on December 22, allowing it to pass. Several countries, for example, Bolivia, have severed links with Israel. South Africa has recently accused Israel at the International Court of Justice (ICJ) of committing genocide in Gaza and the ICJ has concluded that the charge of genocide against Israel is plausible, or more accurately that Gazans had plausible rights against genocide under the Genocide Convention and that these were being concretely threatened by Israel [28]. Egypt, one of Israel's closest allies in the Middle East, has joined the South African case against Israel [29]. In late March 2024, despite being denied entry to Gaza, the Special Rapporteur on the situation of human rights in the Palestinian territory occupied since 1967 to the Human Rights Council has concluded that Israel is committing genocide in Gaza [30].

In response to past and present accusations, Israeli officials have claimed to be abiding by IHL and Israel has indeed been constrained—at least to some extent—by it [16]. The Geneva Conventions are international treaties that have been universally ratified. Particularly the Protocol I Additional to the Conventions is considered, at least in part, customary IHL that dictates the rules of just war. This means that IHL applies to all states and parties to an armed conflict regardless of whether they ratified it.

The IV Geneva Convention governs the conduct of warring parties toward civilians. It most fundamentally and explicitly relies on “The principle of respect for human personality” [31], which applies to all humans qua humans:

it is concerned with people, not as soldiers but simply as human beings, without regard to their uniform, their allegiance, their race or their beliefs, without regard even to any obligations which the authority on which they depend may have assumed in their name or in their behalf. Wounded or sick, they are entitled as such to the care and aid which the respect for human personality enjoins [31, p. 27].

Israel ratified the IV Geneva Convention in 1952 but has argued that it does not apply to the Occupied Palestinian Territory (oPt). International legal consensus, however, maintains that the IV Geneva Convention does apply there [32]. Usually, this means that ethical and legal opposition to Israeli policies and interventions against Palestinians could rely on the Convention [33]. The case of hospitals in Gaza, however, may be an exception, as their immunity is said (by Israel and the supporters of its conduct in Gaza, including healthcare professionals and professional ethicists, as mentioned above) to be forfeited according to Article 19 of the IV Geneva Convention. The following critical analysis explains why this might *appear* to be the case and argues that the article should be revised to better protect hospitals and patients. The analysis further argues that even in its current form, closer scrutiny of Article 19 reveals that it does not apply to hospitals in Gaza, meaning that the appearance here is indeed a mere appearance. In so doing, this paper joins other calls to increase protection of healthcare facilities [1]; for instance, by banning the use of explosives in their vicinity [34].

Before turning to the analysis, the two tests of necessity and proportionality should be mentioned. Understanding these is important to any strategy taken to ensure justice in war—even the bioethical analysis mentioned above emphasizes that the principles advocated only apply in the long run, after these two tests have been employed [17]. According to the United Nations (UN) Charter, states are only allowed to engage in aggression toward other states for self‐defence or general security (states also have the obligation to stop genocide, under the Convention on the Prevention and Punishment of the Crime of Genocide). Recognizing that civilians would necessarily be harmed during armed conflicts, Article 57 of the Protocol Additional to the Geneva Conventions of 12 August 1949, and relating to the Protection of Victims of International Armed Conflicts (Protocol I), outlines two tests for the lawfulness of an attack: necessity and proportionality. The two tests have traditionally been more relevant for *jus ad bellum*, meaning rules pertaining to the decision to engage in war, but they may be considered for *jus in bello*, referring to rules governing the conduct in war [35]. According to the necessity test, an attack is only lawful if it is necessary to achieve the military objective. According to the test of proportionality,

an attack shall be cancelled or suspended if it becomes apparent that the objective is not a military one or is subject to special protection or that the attack may be expected to cause incidental loss of civilian life, injury to civilians, damage to civilian objects, or a combination thereof, which would be excessive in relation to the concrete and direct military advantage anticipated [36].

Under the proportionality test, or really before actually applying the test, an attacker is obligated to first evaluate whether a target has special protection, that is, whether it is a lawful target. Second, an attacker needs to evaluate whether the expected civilian harm, meaning the expected harm to protected targets, is excessive in relation to the expected military gain. If an attacker fails to show that the objective has lost its special protection, then no amount of gain is sufficient to justify the attack. At the same time, the proportionality test also means that an attacker may not be justified in targeting a civilian target such as a hospital even if it did lose its special protection, because of the expected collateral damage to protected civilians or civilian infrastructure around the hospital.

From a philosophical perspective, this moral prioritization of considerations relating to “special protection” status, or rather of the traits that confer special protection and their meaning, over the tests of proportionality and necessity (utility, in his words) was expressed long ago by Michael Walzer:

But though the limits of utility and proportionality are very important, they do not exhaust the war convention; indeed, they don't explain the most critical of the judgments we make of soldiers and their generals. If they did, moral life in wartime would be a great deal easier than it is. The war convention invites soldiers to calculate costs and benefits only up to a point, and at that point it establishes a series of clearcut rules—moral fortifications, so to speak, that can be stormed only at great moral cost [24, p. 161 in electronic version].

There is more, then, to *jus in bello* than the tests of necessity and proportionality.

## 3 On the Special Protection to Hospitals

This section examines whether the IV Geneva Convention corresponds to our sense of right. One way to do this is to make a positive case for the moral sacredness of hospitals as safe havens, as shelters for the most vulnerable. In short, a case could be made to explain why hospitals are morally special. Such a case would have to either argue that hospitals are special while other institutions, such as schools, are not, or that the special moral status of hospitals and the special moral status of other institutions are not mutually exclusive. This task is reserved for subsequent work.

Here, a negative case will be made, arguing that the IV Geneva Convention is hardly defensible from an ethical perspective, as its exceptions to the rule of immunity of hospitals are too wide.

Article 18 of the IV Geneva Convention grants special protection—or near absolute immunity— to civilian hospitals:

Civilian hospitals organized to give care to the wounded and sick, the infirm and maternity cases, may in no circumstances be the object of attack, but shall at all times be respected and protected by the Parties to the conflict [31, p. 141].

Article 19 of the IV Geneva Convention qualifies this immunity:

The protection to which civilian hospitals are entitled shall not cease unless they are used to commit, outside their humanitarian duties, acts harmful to the enemy. Protection may, however, cease only after due warning has been given, naming, in all appropriate cases, a reasonable time limit, and after such warning has remained unheeded.

The International Committee of the Red Cross (ICRC) has added commentaries to explain and elaborate on the various Geneva Conventions. Written by members of the ICRC at different times, these commentaries are not legally binding in the same way the Geneva Conventions are but represent an authoritative explanation and justification of the content and spirit of the Conventions. The Commentary to Article 19 acknowledges that “acts harmful to the enemy” is vague, explaining that,

Such harmful acts would, for example, include the use of a hospital as a shelter for able‐bodied combatants or fugitives, as an arms or ammunition store, as a military observation post, or as a centre for liaison with fighting troops [31, p. 154].

This explanation, however, is unsatisfactory. Surely a morally significant difference exists between a concrete case of self‐defense, as in the case of a hospital sheltering an able‐bodied combatant who is concretely threatening the other party or a hospital serving as a “military observation post,” on the one hand, and a “center for liaison” on the other. Intuitively, the following cases are morally distinct:

A. While risking patients of party Y, a soldier or an officer of party X targets a combatant of party Y who is concretely threatening party X.
B. While risking patients of party Y, a soldier or an officer or a government of party X targets a structure suspected to serve as a center of operation by combatants of party Y who devise and execute plans to harm soldiers and/or civilians of party X.

In both cases, the condition of culpability applies: the lives and bodily integrity of a civilian victim or victims are threatened by an attacker who consequently forfeits their right to life and/or bodily integrity [37].

The threat in case A, however, is much more urgent, and the effects of the counterstrike are much more likely to prevent members of party X from being harmed. This is a strict case of self‐defense—elsewhere defined as a case of villainous aggressor [38]—or an immediate defense of one's own. The strongest philosophical case to justify self‐defense is that persons are entitled to life and to be free from risk to life and bodily integrity, or in other words, that persons have a right to life and bodily integrity:

For what does it mean to say that someone has a right to life? To say that is to recognize a fellow creature, who is not threatening me, whose activities have the savor of peace and camaraderie, whose person is as valuable as my own [24, p. 173].

This right means that others cannot simply kill you without proper justification. If persons are made to face a risk of death, they are justified in annulling it, even if it means killing another person [38]. Hence, neutralizing a combatant who poses an immediate risk to you or your own virtually annuls that risk.

Case B is less urgent and less decisive. The planned attack might be imminent, but the exact timing is probably unknown and, in any case, less urgent than the attack in case A. While destroying a military headquarters probably reduces the chances of executing a successful planned attack, it does not eliminate it, as surviving officers and soldiers may still carry on the attack, albeit in a less synchronized manner. This means that even if an attack is imminent, destroying the headquarters of party Y that is planning the attack would not necessarily abort it, as individuals may still act on their own. This, in fact, was the case during the Cold War, when American and Soviet submarines were instructed to attack the enemy party if communication with their headquarters suddenly ceased, raising suspicion that they had been attacked. The urgency of the risk, then, and the probability of annulling it, are the decisive factors here that make a morally significant difference. Case A, then, is more easily justified from an ethical perspective than case B.

Granted, focusing on the urgency of the risk and the ability to annul it reflects a prioritization of these over the potential gravity of the combatants' attack. Arguably, one combatant of party Y shooting directly at party X may cause a limited degree of harm, while several officers sitting in an office (or tunnel) may cause a much greater harm in terms of degrees of casualties or injuries, locations of a higher political and military strategic importance, and the coordination of several simultaneous attacks in different locations. Such prioritization of urgency and potential of prevention over gravity of risk in this case is justified for two reasons: First, again, because neutralizing these officers does not necessarily mean that the well‐orchestrated attack would be prevented. Second, in real life we rarely know whether this kind of complex planning is occurring in a specific location and point of time, and so practically, justifying an attack on a hospital that serves as a “center for liaison” seems cumbersome.

The main motivation driving the moral difference between the two cases is not military necessity or the forfeiture of the right to life and bodily integrity of the attackers of party Y, or their culpability. Rather, it is the right to life and bodily integrity of the victims or soldiers of party X: in case A their right to life and bodily integrity would surely be preserved; in case B their right to life and bodily integrity may or may not be preserved.

Adding another layer to the critique, even the moral permissibility of killing civilians of party Y in case A should be scrutinized. Consider a hostage scenario, where a kidnaper points a gun at you from behind an innocent civilian. Killing that kidnaper is justified as self‐defense. But what if the only way to kill the kidnaper and reduce the risk to yourself entails killing the hostage? The defense of double effect obviously may be used in this case, as the intention is to solely neutralize the kidnaper; the death of the hostage, while foreseeable and regrettable, is merely incidental.

Yet, it is still morally impermissible or at least morally questionable to kill a hostage of one's own nationality even in such cases of immediate risk, meaning even if it is clearly done as self‐defense. Why? Because first, the right to take action against an attacker is not absolute, and second, because the hostage has an interest in being alive and thus a right to life as well, you are not justified in violating that right even with the price of your own life [39]. If the risk of harming the hostage is high, then you plausibly should simply let the kidnaper shoot you. In more formal language, the right to life and bodily integrity of the hostage is not outweighed by your right to self‐defense because the hostage is not the one who is threatening you. Only the one threatening you (or who is actively collaborating with those who threaten you) loses their right to life and bodily integrity.

If this reasoning is compelling, there is no obvious reason why it should apply differently in the case of innocent, unarmed, and vulnerable patients of other nationalities [40]. In other words, justifying the harm toward innocent civilians of party Y by soldiers of party X in case A is ethically akin to justifying the harm toward innocent civilians of party X by soldiers of party X. At the very least, the burden of justification here is extremely heavy. For the purposes of this paper, however, engaging in this much more complex and controversial dilemma is not necessary.

Taking stock, a hospital from which a belligerent is firing at you or at your co‐patriots may legitimately and reasonably lose its legal immunity under IHL. A hospital that merely serves as a command center may also lose its legal immunity under IHL. This is unreasonable. The criteria to evaluate reasonableness in this case are the urgency of the threat and the prevention potential. These are then applied to evaluate different outcomes regarding the potential victim's right to life and bodily integrity, grounding a right to self‐defense. Furthermore, targeting a hospital, even if it is done legitimately, carries an extremely high burden of proof because of the risk to innocent patients, in the same way as harming a co‐patriot hostage is.

## 4 Re‐establishing Special Protection to Hospitals in Gaza

One commentator has similarly called to increase the protection of hospitals under IHL, because in his reading of Article 19, “health‐care facilities can be targeted after a warning and after enough time has been given to allow for the evacuation of patients and staff” [34]. Even in its existing version, however, Article 19 does NOT back the Israeli bombing of hospitals in Gaza. This only becomes apparent upon closer scrutiny. The Commentary further qualifies Article 19 by clarifying the conditions necessary for its implementation:

“The enemy will, therefore, warn the hospital to put an end to the harmful acts and will fix a time limit, on the expiry of which he may attack if the warning has not been heeded. The period of respite is not specified. All that is said is that it must be reasonable. How is it to be determined? It will obviously vary according to the particular case. One thing is certain, however. It must be long enough to allow the unlawful acts to be stopped or for the hospital patients to be removed to a place of safety” [31].

Israel has, in fact, warned Al‐Shifa hospital and made its intentions clear to Palestinians and the international community. In light of the alleged fact that the political arm of Hamas was unaware of the plan and its execution by the military arm of Hamas, Hamas as a whole has proven itself to be an unreasonable or rather a dysfunctional organization and it thus cannot be expected to evacuate its headquarters to protect patients. For Article 19 to be applied, Israel must ensure sufficient time for “hospital patients to be removed to a place of safety.” But where in Gaza does that exist? Since October 7, Israel has engaged in 670 Israeli attacks on healthcare personnel and institutions, resulting in the death of 886 and injury of 1355 people, as well as in the damaging of 33 hospitals and 133 ambulances [6]. More than 61 Healthcare workers have been detained or arrested [41]. These, in addition to the grave lack of potable water and electricity, mean that Gazan patients do not have any healthcare facility to provide a safe haven, and even if there were, there are no adequate medical transportation measures to transfer dialysis patients, those who are gravely wounded, and other immobile patients. Based on this interpretation, Article 19 actually protects patients at Al‐Shifa and other hospitals in Gaza. Importantly, this would also have to be taken into account in case A above, where hospitals indeed lose their immunity, but attackers still need to be held accountable based on the proportionality test.

## 5 Response to Actual and Potential Objections

Actual and potential objections may be divided into two kinds, one regarding the content and conclusions of the discussion above, and one regarding the raison d'etre of the paper more generally.

At least three objections concerning the content and conclusions of the discussion may be raised. A first objection that may, and has in fact been raised, challenges my assumption above that at the time of writing, there was no safe place in Gaza to which patients could be transported. Since there were indeed safe places for patients, it is not the case that hospitals retained their immunity. My response is twofold: First, this is a primarily empirical question (but also subjective, as patients also need to evaluate the place they are being transported to as safe). There is surely great malevolent deception employed both by Hamas and the Israeli and other governments, as well as fake news in news and social media, that deciphering the current reality in Gaza may be challenging. If, in some way, it turns out that there were indeed safe places and that transportation measures were available and never threatened, then indeed this would be determined by this argument. Second, the burden of proof showing that patients could have indeed been transferred to save havens rests on Israel, but as yet, the Israeli government has not provided any such proof.

A second objection may be lodged against my prioritization of the urgency and prevention potential criteria over the gravity of harm criterion. Above, I reasoned that they should receive priority first because the main value that should be protected is the non‐combatant's right to life. Annulling the certain, immediate risk is much more likely to preserve that right. The criteria of urgency of harm and prevention potential should also receive priority simply because it is practically difficult to know whether and when some meeting in a military command center would result in an attack. Both of these, however, are admittedly weak arguments. Remaining within the realm of utilitarianism, an interlocutor may respond that, exactly because the underlying value is a human right to life and bodily integrity, saving the most lives matters more than saving one life, even if the probability of the former is lower compared to the latter. Ultimately, the deliberation here would likely end up being a debate between two maxims without a possibility for deeper justification: the importance of the urgency of harm and prevention potential on the one hand, and the importance of the collective gravity of harm.

A third objection relates to the future application of a revised, more restrictive Article 19. Imagine that Article 19 is revised so that hospitals that are merely used as a command center do not lose their immunity. Imagine even that, being compelled by my hostage scenario, articles 18 and 19 note provide a stronger immunity to hospitals even if a belligerent is actively shooting at you. This may save lives and better protect the right to life and bodily integrity among the patients at the hospital, but it would also increase the risk for combatants and non‐combatants who may suffer the results of a terror attack on their soil, for instance. The response to this objection is simply biting the bullet—indeed, more of our own party's combatants and noncombatants may be harmed as a result, for example, in a wide‐scale terrorist attack that takes place, but this is the price we need to pay to uphold our sense of the right.

A fourth objection that warrants a response is a general one about the motivation for this paper, whether it should justifiably take up space in an overly burdened bioethics journal. Indeed, an editor of a preeminent bioethics journal has recently used the following reasoning in rejecting an earlier version of the current paper: “While this is a well‐written manuscript, our biggest concern is that we have a hard time believing that anyone amongst the Bioethics community would disagree with the conclusion about hospitals.”

At least two responses are in order here. First, publications in a specific discipline are not meant solely for that discipline. Hopefully, in the age of the World Wide Web, disciplines can interact with and benefit from one another at least by occasionally reading each other's work. Moreover, the bioethics or applied ethics literature should be used, made accessible, and promoted as exactly the place to go for readers interested in concrete healthcare dilemmas, such as the bombardment of hospitals and, in fact, the violent curtailment of access to healthcare. In other words, deliberating the ethics and legality of hospital bombardment in the bioethics or applied ethics literature is worthwhile even if all ethicists agree that it is wrong. It is worthwhile because it provides (hopefully) authoritative resources and guidance to healthcare professionals and legal experts who examine this dilemma or who express misguided normative statements. For instance, a group of Israeli healthcare professionals has indeed argued that Israel is justified in targeting hospitals in Gaza and thus prioritizing the safety of Israeli soldiers over unarmed Palestinian civilians [42].

Two, it is simply not the case that all bioethicists “would disagree with the conclusion about hospitals.” Many Israeli bioethicists (as well as medical educators and medical humanists) have supported the bombardment of hospitals in the ongoing war in Gaza. Already in 2006, Michael Gross had defended the Israeli bombardment of Palestinian hospitals:

Israel presents two different arguments in response to charges of violating medical neutrality and obstructing medical care. One argument reaches to the conventional and reciprocal nature of medical immunity and suggests that once one side violates neutrality, the other side is no longer bound to respect it. The other argument accepts the overwhelming importance of medical immunity but argues that the obligation to respect medical neutrality is not absolute and may be overridden by military necessity in exceptional cases. Each of these arguments is already part of international law. Hospitals and medical facilities lose their protected status if used for hostile purposes. [4, p. 191]

Alas, as shown above, this interpretation of the idea of medical neutrality and the immunity of hospitals granted under IHL was lazy and superficial even then, and in any case, does not apply to the current situation in Gaza. Indeed, the immunity of hospitals is not absolute under IHL, but the burden of proof on the part of the attacking army is much higher than what Gross makes it to be. Similarly, reciprocity in the context of IHL does not mean what Gross thinks it means. Rather, Reciprocity in the context of IHL first means that only countries that have signed it are protected by it, and only the articles that were signed without reservations apply to the signing party. This is simply the nature of international treaties. Second, and more relevant to us here, reciprocity in the context of IHL might mean that once one party to the conflict violates IHL, then the other party is allowed to do so too. Meaning, once Palestinians have violated the neutrality of their hospitals, Israel is justified in doing so as well. IHL is explicitly against this kind of reciprocity [43].

A fifth potential objection that is generally about any discussion of just war and IHL in the context of Gaza questions the practical relevance of this paper, even if it presents a compelling argument. Regardless of the (philosophically weak) justification given by Gross to Israel in its targeting of hospitals, the threshold to legitimize the targeting of hospitals under IHL is already extremely high [44]. States that follow IHL will find it extremely challenging to justify such targeting. On the contrary, states that have not demonstrated any willingness or intention to adhere to IHL are likely to continue their rogue conduct regardless of the philosophical grounding and justification of IHL. Since its very inception, Israeli governments have consistently violated international law [45, 46], and are unlikely to care much about the present discussion. Unfortunately, I have no response to the counterargument other than this. One can choose optimism or despair; I choose optimism:

…if we succumb to despair we will help ensure that the worst will happen. And if we grasp the hopes that exist and work to make the best use of them, there might be a better world. Not much of a choice. [47]

## 6 Conclusion

Currently, under IHL and certain conditions, the immunity of hospitals may be waived. The Israeli government has argued that these conditions have indeed been met, and hence it could legitimately target hospitals (along with ambulances and healthcare personnel).

This article disagrees with this claim. Once the exact manner in which such conditions may be met is clarified, it becomes clear that these conditions have not been met in Gaza. Namely, patients have no safe place to go to seek shelter and continuity of care. For future reference, this article argues that the language of Article 19 should be revised to clarify that hospitals may lose their immunity only if patients can be evacuated to a place of safety.

This paper further argues that Article 19 of the IV Geneva Convention should be revised to protect patients better and uphold the immunity of hospitals in all cases except when there is a concrete threat from within the hospital. Only in these cases will hospitals legitimately lose their immunity, and a consideration of proportionality would then apply. Otherwise, hospitals maintain immunity, obviating the need for a proper proportionality analysis. Even in cases of concrete threat, however, the burden of justification is extremely heavy, potentially in a similar fashion to harming hostages of one's own nationality to neutralize a concrete threat.

This paper only makes a negative case for the increased protection of hospitals in Gaza. A more comprehensive account will also make a positive case, articulating what makes hospitals so special that they warrant special protection even at times of war.

## Data Availability

Data sharing is not applicable to this article as no data sets were generated or analyzed during the current study.

## Endnotes

1 I emphasize “for instance” because I am not committed to the moral ontology that Daniels seems to be committed to with his WRE, meaning the impossibility of an objective or common morality. Instead, for the purposes of this paper, I adopt WRE merely as a procedural tool to understand and engage in moral reasoning. I could have done the same using the idea of a common morality. This is indeed the only way in which Daniels' WRE can be aligned with Dworkin's foundationalism.

## References

[1] L. Rubenstein , Perilous Medicine (Columbia University Press, 2021).

[2] Z. Lederman , “Together We Lived, and Alone You Died: Loneliness and Solidarity in Gaza,” Developing World Bioethics 21 (2021): 17–24. 32573955 10.1111/dewb.12272

[3] Z. Lederman and S. Lederman , “The Responsilbity of Bioethicists—The Case Study of Yemen,” Bioethics 39 (2025): 67–75. 37917807 10.1111/bioe.13231 PMC11657458

[4] M. L. Gross , Bioethics and Armed Conflict: Moral Dilemmas of Medicine and War (MIT Press, 2006).

[5] R. Hoval , “There Are Two Abductees You haven't Heard of”: An Interview With the One Following Them, in *The Hottest Place in Hell* (2024), https://www.ha-makom.co.il/post-revital-yuval.

[6] World Health Organization , “oPt Emergency Situation Update Issue 56” (2025), https://reliefweb.int/report/occupied-palestinian-territory/opt-emergency-situation-update-56-7-oct-2023-28-feb-2025.

[7] World Health Organization, “oPt Emergency Situation Update Issue 30” (2024), https://reliefweb.int/report/occupied-palestinian-territory/opt-emergency-situation-update-30-7-oct-2023-8-may-2024-1600.

[8] “Israeli Tanks Surround Indonesian Hospital in Gaza, Heavy Fighting Breaks Out,” *Sydney Morning Tribune*, 2023, https://www.smh.com.au/world/middle-east/israeli-tanks-surround-indonesian-hospital-in-gaza-heavy-fighting-breaks-out-20231121-p5elhv.html.

[9] Al‐Shifa Hospital , “Annihilation and Resilience,” Gaza, *Al Jazeera*, accessed March 2025, https://www.aljazeera.com/program/featured-documentaries/2024/10/13/al-shifa-hospital-annihilation-and-resilience.

[10] C. Danner , “What Has Israel Found Inside Gaza's Al‐Shifa Hospital?,” *Intelligencer*, 2023, https://nymag.com/intelligencer/article/what-has-israel-found-inside-gaza-al-shifa-hospital.html.

[11] B. Osgood , A. Harb , and U. Siddiqui , “Israel's War on Gaza Updates: Patients Flee Nasser Hospital Under Fire,” *Al Jazeera*, 2024, https://www.aljazeera.com/news/liveblog/2024/2/15/israels-war-on-gaza-live-four-dead-as-israel-hits-city-in-lebanons-south.

[12] “Video,” Amanpour & Company, November 20, 2023, https://www.nytimes.com/2024/01/02/us/politics/gaza-hospital-hamas.html.

[13] J. E. Barnes , “Hamas Used Gaza Hospital as a Command Center, U.S. Intelligence Says,” *The New York Times*, 2024, https://www.nytimes.com/2024/01/02/us/politics/gaza-hospital-hamas.html#:~:text=U.S.%20spy%20agencies%20believe%20that,American%20intelligence%20declassified%20on%20Tuesday.

[14] D. Y. Kovovitch , “In the Tunnels Under Shifa, the Question of Whether Hamas Acted From Here Becomes Superfluous,” *Ha'aretz*, 2023, https://www.haaretz.co.il/news/politics/2023-11-22/ty-article-magazine/.premium/0000018b-f818-d783-a3df-f81ad61d0000.

[15] E. Fabian , “Gaza Hospital Director Admits Hamas Used Medical Complex as Operational Hub,” *The Times of Israel*, 2023, https://www.timesofisrael.com/gaza-hospital-director-admits-hamas-used-medical-complex-as-operational-hub/.

[16] N. Perugini and N. Gordon , “Medical Lawfare: The Nakba and Israel's Attacks on Palestinian Healthcare,” Journal of Palestine Studies 53 (2024): 68–91.

[17] N. S. Jecker , C. Atuire , V. Ravitsky , K. Behrens , and M. Ghaly , “War, Bioethics, and Public Health,” American Journal of Bioethics 25, no. 5 (2025): 106–120. 10.1080/15265161.2024.2377118 39037719

[18] H. Slim , Solferino 21_Warfare Civilians and Humanitarians in the Twenty First Century (Oxford University Press, 2022).

[19] Z. Lederman , S. Lederman , and N. Davidovitch , “Medical Ethics at War: Is Israel Obligated to Treat Hamas Combatants?,” Journal of Jewish Ethics 9, no. 2 (2023): 184–208, https://scholarlypublishingcollective.org/psup/jewish-ethics/article-abstract/9/2/184/400401/Medical-Ethics-at-War-Is-Israel-Obligated-to-Treat?redirectedFrom=fulltext.

[20] R. Dworkin , Taking Rights Seriously (Harvard University Press, 1978).

[21] N. Daniels , “Wide Reflective Equilibrium and Theory Acceptance in Ethics,” Journal of Philosophy 76 (1979): 256–282.

[22] L. May , Crimes Against Humanity (Cambridge University Press, 2005).

[23] D. Luban , “A Theory of Crimes Against Humanity,” Yale Journal of International Law 29 (2004): 85–167.

[24] M. Walzer , Just and Unjust Wars: A Moral Argument with Historical Illustrations, 5th ed. (Basic Books), 2015 (1977), 163–164.

[25] The Federal Government, “Germany Stands by Israel—and Is Seeking to Bring About a De‐Escalation,” 2023.

[26] N. Erakat , Justice for Some: Law and the Question of Palestine (Stanford University Press, 2019).

[27] Lederman , “United Nations General Assembly. Report of the Independent International Commission of Inquiry on the Occupied Palestinian Territory, including East Jerusalem, and Israel, Rubenstein,” 2022.

[28] International Court of Justice, “Application of the Convention on the Prevention and Punishment of the Crime of Genocide in the Gaza Strip (South Africa v. Israel),” 2024.

[29] “Why Egypt Joining ICJ Case Against Israel is ‘Unprecedented’,” Israel War on Gaza, *Al Jazeera*, accessed May 2024.

[30] “Anatomy of a Genocide. Report of the Special Rapporteur on the Situation of Human Rights in the Palestinian Territories Occupied Since 1967, Francesca Albanese,” 2024.

[31] J. S. Pictet , “Commentary to The IV Geneva Convention,” Geneva, 1958, p. 26.

[32] United Nations, “The Question of The Observance of the Fourth Geneva Convention of 1949 in Gaza and The West bank Including Jerusalem Occupied by Israel in June 1967,” 1979.

[33] Z. Lederman , E. Shepp , and S. Lederman , “Is Israel Its Brother's Keeper? Responsibility and Solidarity in the Israeli–Palestinian Conflict,” Public Health Ethics 11 (2018): 103–120.

[34] M. R. van der Heijden , “Attacks on Hospitals: Current Legal Protections Are Insufficient,” Lancet 402 (2023): 2293–2294. 38048791 10.1016/S0140-6736(23)02632-6

[35] J. Gardam , Necessity, Proportionality and the Use of Force by States (Cambridge University Press, 2004).

[36] “Protocol Additional to the Geneva Conventions of 12 August 1949, and relating to the Protection of Victims of International Armed Conflicts (Protocol I),” 1977.

[37] Self‐Defense (Stanford Encyclopedia of Philosophy), accessed February 2024.

[38] J. J. Thomson , “Self‐Defense,” Philosophy & Public Affairs 20 (1991): 283–310.

[39] U. Steinhoff , On the Ethics of War and Terrorism (Oxford University Press, 2007).

[40] J. Mcmahan , Killing in War (Oxford University Press, 2009).

[41] World Health Organization, “oPt Emergency Situation Update Issue 22,” 2024.

[42] עקרון הצדק ברפואה והמלחמה בעזה ‐ העוקץ (haokets.org), accessed May 2024. (Hebrew).

[43] J. de Preux , “The Geneva Conventions and Reciprocity,” International Review of the Red Cross 25 (1985): 25–29.

[44] “The Protection of Hospitals During Armed Conflicts: What the Law Says,” ICRC, accessed May 2024.

[45] R. K. Erakat , The Hundred Years' War on Palestine (Picador Metropolitan Books, 2021).

[46] N Chomsky , Fateful Triangle (Sound End Press, 1999).

[47] N. Chomsky , Optimism Over Despair—On Capitalism, Empire, and Social Change (Kindle Edition, 2017).

